# Residual energy use and energy efficiency improvement of European supermarket facilities during the post-COVID and energy crisis period

**DOI:** 10.1016/j.heliyon.2024.e29781

**Published:** 2024-04-17

**Authors:** Juan Carlos Ríos-Fernández, Juan Manuel González-Caballín, Andrés Meana-Fernández, Antonio José Gutiérrez-Trashorras

**Affiliations:** University of Oviedo, EDZE (Energy), Campus de Viesques, 33203, Gijón, Asturias, Spain

**Keywords:** Supermarkets, Energy saving, Greenhouse gas emission reduction, Waste heat recovery, Post-Covid, Energy crisis

## Abstract

Supermarkets are significant consumers of electricity and contribute to the generation of associated pollutant emissions. This will help to mitigate the impact of increased energy costs on the prices of products sold in supermarkets. Therefore, it is essential to reduce energy consumption, starting with the equipment that consumes the most electricity, such as refrigeration, and using the residual thermal energy generated in supermarkets. This paper discusses the impact of rising energy costs in the post-Covid era and during the energy crisis. It evaluates the environmental and energy benefits of implementing energy improvements and utilizing residual energy in real supermarkets. The analysis takes into account the socio-economic characteristics of the EU-27 countries, which affect the economic feasibility of these measures. This would prevent the release of 122 tons of CO_2_ per year for each supermarket, resulting in energy savings of around 70 % or 305 kWh/m^2^. The required investments would have a payback period of 4 years.

## Highlights

• Improvement of energy efficiency to mitigate energy costs in supermarkets.
• Post-Covid and the energy crisis modify energy expenditures in European supermarkets.
• Residual thermal energy from refrigeration to reduce HVAC energy consumption.
• Waste heat from bakery ovens is used to heat water and reduce energy consumption.

## Abbreviations

*CNMC*: Spanish National Markets and Competition Commission
*COP*: Coefficient of performance
*CO*_*2*_: Carbon dioxide
*CO*_*2eq*_: Carbon dioxide equivalent
*CPI*: Consumer price index
*DHW*: Domestic hot water
*EU*: European Union
*EU-27*: European Union-27
*GWP*: Global Warming Potential
*HVAC*: Heating, ventilation and air conditioning
*IDAE*: Instituto para la diversificación y ahorro de la energía
*IWERS*: Integrated waste energy recovery system
*LNG*: Liquefied natural gas
*MWh*: Megawatt hour
*m*^*2*^: Square meter
*t*: Ton
*TWh*: Terawatt Hour
*€*: Euro

## 1 Introduction

The COVID-19 pandemic caused a global economic slowdown, directly affecting European countries in 2020 [1]. As energy demand began to recover, a natural gas shortage in 2021 [2], compounded by cuts in nuclear energy supply and adverse weather conditions reducing wind in Europe, led to an energy crisis. The armed conflict between Russia and Ukraine has led to economic sanctions from the European Union (EU) against Russia [[3], [4], [5]]. As a result, there has been a scarcity in the supply of Russian gas to Europe for its combined cycle plants, which has led to an increase in the cost of energy during 2022. Furthermore, the utilization of lignite coal and hydrocarbons has become necessary due to the limited generation of wind and solar energy, resulting in increased costs associated with carbon dioxide (CO_2_) emissions permits [6]. In the first half of October 2021, the average electricity price reached a new record of 202.77 euros (€) per megawatt hour (MWh), which is 30 % higher than the previous month's price of 156 €/MWh in September 2021 [7]. The current energy crisis has resulted in higher product costs at supermarkets and decreased profits for commercial companies [[8], [9], [10]]. According to the World Bank, energy prices are expected to continue rising throughout 2023, with no short-term relief in sight [11]. As a result, Spanish supermarkets paid 240 €/MWh for electricity during the first quarter of 2022, which is 20 % higher than the EU-27 (1) average [12]. Four years ago, the average price paid by establishments in Spain was more than nine percentage points below the EU27 average. Thus, the average price during 2018 was 158 €/MWh. France paid an average of 155 €/MWh, Germany paid 224 €/MWh, Italy paid 198 €/MWh, and the EU27 paid an average of 172 €/MWh [7]. In 2008, Spain paid only 130 €/MWh for electricity in its supermarkets, while the EU average was 160 €/MWh [13]. From the first quarter of 2008 to the first quarter of 2022, the price of electricity in Spanish supermarkets increased by 66 %, compared to 36 % in the EU-27 and 39 % in the Eurozone (2) [7]. In March 2022, the flash estimate of the Consumer Price Index (CPI) (3) in Spain showed an annual change of 9.8 %, which is more than two percentage points higher than the previous month's figure. The leading indicator of Core Inflation (4) increased by 0.4 percentage points to 3.4 % in 2022 [14]. To achieve the ambitious goal of reducing polluting emissions into the atmosphere, it is crucial to minimize energy consumption in non-domestic buildings, such as supermarkets [15,16].

(1) The EU-27 comprises Germany, Austria, Belgium, Bulgaria, Croatia, Denmark, Slovakia, Slovenia, Spain, Estonia, Finland, France, Greece, Hungary, Ireland, Italy, Latvia, Lithuania, Luxembourg, Malta, The Netherlands, Poland, Portugal, the Czech Republic, Romania, Sweden, and Cyprus.
(2) The Eurozone comprises Germany, Austria, Belgium, Slovakia, Slovenia, Spain, Estonia, Finland, France, Greece, Ireland, Italy, Latvia, Lithuania, Luxembourg, Malta, the Netherlands, Portugal, and Cyprus.
(3) The Consumer Price Index (CPI) is a statistical measure of the evolution of the prices of goods and services consumed by the population that resides in family dwellings in Spain.
(4) Core Inflation is the inflation reflected by the consumer price index when it does not consider either energy products or unprocessed food products.

The COVID-19 pandemic and the current energy crisis have significantly impacted the operations and energy consumption patterns of the food retail sector, particularly in European supermarket facilities [1]. These events have highlighted the need for a deeper understanding of waste energy use and the identification of effective measures to improve energy efficiency in this critical industry. Existing research has primarily focused on the energy consumption and efficiency of supermarket facilities during normal operating conditions [17]. However, the post-Covid period and the energy crisis have introduced new challenges and dynamics that require further research. Changes in consumer behavior, supply chain disruptions, and energy price volatility have contributed to a shifting energy consumption landscape for European supermarkets.

This study aims to address the challenges and evaluate waste energy use in European supermarket facilities during the post-COVID and energy crisis period. The research provides information on long-term energy consumption trends and identifies opportunities for improvement in energy efficiency. The research sights to help the industry address current challenges and achieve sustainable energy savings by exploring and evaluating effective strategies that are feasible. It assesses the economic and environmental benefits of implementing energy efficiency measures, quantifying potential cost savings and environmental impacts. This can provide a strong business case for supermarket operators to invest in energy-efficient technologies and practices.

Developing a framework for the continuous management and optimization of energy in European supermarket facilities can enable supermarket operators to systematically monitor, analyze, and optimize their energy consumption over time, ensuring long-term sustainability. The findings of this study will contribute to broader research on energy efficiency in the grocery retail sector, particularly in the context of disruptive events such as the COVID-19 pandemic and energy crises. The generated insights and recommendations can assist European supermarket operators in making informed decisions, enhancing their energy performance, and improving the overall sustainability of the industry.

The paper's original contribution is the calculation of energy savings during the post-COVID and energy crisis period in the most common EU supermarket model. This period was characterized by uncertainty in energy consumption and constant price increases. Previous research did not jointly analyze the improvements and uses of energy presented in this study, nor did it focus on this specific and significant period in history. Secondly, the economic data covers all EU-27 countries. Thirdly, the analysis is novel because it applies to efficiency improvements in all energy-consuming installations and the use of residual energy with quantified methods in a real EU representative supermarket. It is crucial to reduce energy consumption through improvements and innovative methods of harnessing waste energy. Although these aspects may be present in other sectors such as industry, they are uncommon in the retail sector. Therefore, this paper is particularly relevant as it provides data and methods that can be applied to both new facilities and supermarket reforms. Additionally, Spain and Portugal (the Iberian Peninsula) were considered energy islands within Europe. For the first time, this report provides data on the increase in energy prices across all 27 EU countries. The report analyzes the case of Spain in relation to the average energy prices in the EU-27. The results are presented in both technical and economic terms, reflecting the savings in energy and associated reduction in polluting emissions, as well as the payback periods of the economic investments made in the improvements.

### 1.1 Reasons for the increase in energy prices

#### 1.1.1 COVID-19 pandemic

During the 2020–2021 period, the COVID pandemic had a significant impact on Spanish and European society, resulting in a slowdown of economic activity and a subsequent decrease in electricity consumption [18]. Additionally, the pandemic-induced confinement measures and reduction in leisure activities led to a decrease in energy demand. As a result, fuel and energy producers were forced to reduce their production until the pandemic lockdowns were lifted. In 2021, the market quickly recovered with the lifting of some lockdowns and the resumption of commercial activity. However, the production of energy and fuels could not keep up with the demand [[19], [20], [21], [22]]. Additionally, geopolitical factors led to a decrease in gas supply from non-EU countries. Specifically, Spain experienced a reduction in gas availability when the supply from Algeria through the gas pipeline that crossed Morocco was cut off. However, the supply was replaced by chartering liquefied natural gas (LNG) carriers, which made it possible to buy in the global market. This, however, resulted in higher transport costs and a shortage of energy fuels such as gas, leading to increased prices worldwide, particularly in Europe.

#### 1.1.2 EU environmental policies

In recent years, European environmental policies have focused on decarbonizing the energy sector. In 2020, the EU committed to reducing its emissions by at least 55 % by 2030 compared to 1990 levels by signing the Paris Agreement. The EU also signed the Katowice climate package to promote the implementation of the Paris Agreement [23,24]. The price of CO_2_ emission rights has increased. For instance, in Spain, the price of these rights was 24.75 € in January 2020 and 80 € in December 2021 [25]. Furthermore, the shift towards a new energy paradigm in Spain was hastened in 2020 by the closure of most coal-fired power plants. This led to a decrease in energy self-sufficiency from domestic sources. In 2020, other EU countries made significant changes to their energy generation models by phasing out hydrocarbons and coal. This move has exposed them to foreign fuel prices, particularly the cost of natural gas from non-European countries like Russia and Syria.

#### 1.1.3 The price of natural gas

Eurostat reports that natural gas accounts for 20 % of electricity generation in Europe [26]. Furthermore, due to the design of the European electricity market, the price of electricity is largely determined by the price of natural gas [27]. In March 2022, the price of natural gas increased by 25 % in just one year and was multiplied by five at the beginning of the Russian invasion of Ukraine [7]. The increase in gas prices can be attributed to temporary factors such as bottlenecks, reduced production, and investments due to the halt in demand in March 2020, followed by a freezing winter and spring 2021. These factors coincided with a period of very low gas prices before and during the pandemic, resulting in reduced reserves stored in Europe. Furthermore, during the pandemic, China transitioned some of its energy consumption from coal to gas, while Russia limited its gas sales to Europe. Additionally, the United States halted shale gas extraction. As a result, the EU, which heavily relies on imported gas, has experienced a surge in prices. It is worth noting that historically, Russia has been the EU's primary natural gas supplier [28]. Following the Russia-Ukraine-Europe gas disputes of 2006 and 2009, as well as the tensions that arose in the wake of the Ukraine crisis of 2013–2014, the EU aimed to decrease its reliance on Russian natural gas imports [29]. However, as of early 2022, Russia still accounted for approximately 40 % of the EU's gas consumption [30,31]. The price of fuels used in power generation increased significantly due to various factors, including the reduction of Russian oil and gas resulting from the EU sanctions against Russia for the war against Ukraine. This led to a decrease in the availability of fuel worldwide, causing an increase in prices [3,4]. The main factors that affected the price of electricity were the

• electricity production mix available at any given time,
• the price of CO_2_ emission
• rights, the price of
• fossil fuels,
• weather conditions, and the demand for energy by consumers.
• The military conflict between Russia and Ukraine also played a role.

The increase in the price of electricity in Spain and the rest of the EU due to the rise in gas costs should not have had such a significant impact on the final rate for consumers if all these factors were not combined.

### 1.2 The high consumption of electrical energy in supermarkets

A report by the consulting firm Savills states that in 2022, the total stock of food surfaces in Spain reached 16.7 million square meters and 24,522 establishments [32]. This figure includes hypermarkets, supermarkets, discount stores, cash & carry, convenience stores, and specialty stores. Compared to 2019, there was an increase of 1 % in sales room and 8 % in the number of establishments. In 2021, the food sector was the main driver of retail investment in Spain [32]. Table 4 in the annex [[32], [33], [34]] reflects the supermarket formats in Spain during 2020, categorized by size and percentage. These sales area values (m^2^) are similar to those in the rest of Europe, as demonstrated by Tassau et al.'s research [35] and DEFRA's report [36].

Consumption habits have shifted, resulting in an increase in electricity consumption by supermarkets. In the last five years, the food retail sector in Spain has undergone a revolution in the online channel, leading to increased sales. During the first quarter of 2021, the volume increased by almost 600 million euros, representing a 106 % increase [32]. The trend towards consuming refrigerated and frozen foods has resulted in an increase in the number of these products in supermarkets, which in turn requires more energy to cool them [35,37]. Additionally, food retail, particularly supermarkets, has the highest energy consumption in relation to commercial area, primarily due to the need for refrigeration of several products they offer. The energy consumed in a modern supermarket is distributed among various uses, including refrigeration for food storage, lighting, heating, and cooling [37]. Based on the establishment's useful area, electricity consumption values range from 440 to 600 kWh/m^2^·year, depending on the local weather conditions [38]. As a result, modern supermarket companies account for 3 %–5 % of the world's total electricity consumption [39].

In 2011, the percentage of electricity consumption in a typical United Kingdom supermarket depended on various factors, including the supermarket format and the products sold [40]. For the analyzed supermarket model, the results showed that lighting accounted for 15%–25 %, industrial refrigeration accounted for 30%–60 %, and air conditioning, ventilation, and bakery accounted for 25 % of the total electricity consumption. In 2018, Ríos & Roqueñí obtained the following percentages of electricity consumption for a Spanish supermarket with similar characteristics: 29.68 % for lighting, 49.55 % for refrigeration, 8.14 % for heating, ventilation, and air conditioning (HVAC), 9.55 % for bakery, 0.41 % for hot water, 1.61 % for plugs, and 1.06 % for other uses [17]. The data from both investigations are very similar, with small variations due to differences in climatology.

## 2 Materials and methods

The methodology used involved analyzing the energy consumption of a real supermarket in Spain. The study focused on the various areas and facilities within the supermarket that are representative of the EU. The selected supermarket is a typical establishment in terms of surface area, age, and sales areas. The commercial area spans between 1000 and 1500 m^2^ and includes sections for butchers, delicatessens, fishmongers, greengrocers, and bakeries. Additionally, there is a public patio with self-service product sales. According to the consumer market analyzer Veraart Research Group in 2021, the top 10 food retailers in Europe, which comprise the ten most important supermarket companies in Europe, have over 59,000 stores [41]. Supermarket sizes vary depending on the country, region, and store format. The most common size for supermarkets in these companies is around 1000 m^2^ of commercial surface for Aldi Group [42], 1300 m^2^ for Lidl Group [43,44], 1000–1500 m^2^ for Edeca Group [45] and E. Leclerk Group [46], and over 1000 m^2^ for Carrefour Group [47] and Rewe Group [48].

The supermarket is 15 years old, and its facilities have not been renovated since its inauguration. This age is typical of most commercial establishments of this type in Spain and Europe [32]. Energy consumption was measured in areas that could be improved between 2016 and 2017 for 12 months. Subsequently, the supermarket underwent a complete refurbishment, with all facilities being replaced. The energy consumption from 2018 to 2021 was obtained after implementing energy-saving measures during the 2017 reform. To gather information on the supermarket's electricity usage before and after the renovation, individual electric meters were installed in the electrical panels for lighting, industrial refrigeration, air conditioning, ventilation, and in the bakery section with ovens for bakery and pastry production. The electric meters continuously and without interruption measured consumption for ten years, providing daily measurements. This allowed for the calculation of average consumption values for each zone, which are presented in Table 2. The savings resulting from the supermarket reform improvements are listed below.

Equation (1) was used to calculate the yearly electricity savings.

(1) E = B − L

In this study, the variables used are E for electricity savings (kWh), B for the annual electricity consumption of the supermarket before implementing any improvements (kWh), and L for the annual electricity consumption of the supermarket after implementing improvements (kWh).

The difference in consumption was calculated annually by obtaining data on the supermarket's electricity consumption before and after the renovation. Annual CO_2_ emissions savings were calculated using Equation (2).

(2) G = E · P

where, G represents the annual CO_2_ emissions savings (kg CO_2eq_); P represents the carbon footprint (kg CO_2eq_/kWh), as indicated in section 3.2.

Equation (3) was used to calculate the economic savings.

(3) S = E · Z

where, S represents the economic savings (€) and Z represents the energy prices (€/kWh), as indicated in section 3.2.

The results are particularly noteworthy as they reflect the changes in commercial behavior, consumption, energy prices in the post-Covid situation and the energy crisis experienced in Europe. The obtained values were compared with similar analyses carried out by other authors in European supermarkets in the pre-Covid era. A comparative analysis was conducted on the costs of electrical energy consumed in supermarkets over the last 10 years, both in Spain and in the rest of the EU27 countries, was also carried out. This provided a broader perspective on the energy savings and associated reduction in polluting emissions achieved through the presented improvements. Furthermore, the investment in improvements was evaluated and the amortization was calculated.

The formula for calculating the time it takes to recover the cost of an investment, or the simple payback period, is commonly used in all types of improvements. The formula is:

(4) $$A=\frac{C}{F}$$

where, A (year) is the payback period. Here, C (€) represents the cost of applying energy improvements or initial investment, and F (€/year) represents the financial savings achieved with the application of energy improvements or annual cash flows.

The degree of uncertainty may vary depending on the context and factors considered. Uncertainty analysis is crucial when analyzing facility energy efficiency. Building energy assessments must consider various sources of uncertainty, including occupant behavior, thermal properties of the building envelope, and climatic conditions [49]. Modern uncertainty quantification techniques have been widely implemented in various areas of building energy analysis, including model calibration and life cycle assessment [50]. Therefore, it is essential to consider and address these uncertainties to ensure robust and reliable energy efficiency analyses. However, determining the specific numerical value of uncertainty in energy efficiency analyses is not universally standardized and should be based on the characteristics and requirements of each analysis. Bozorgi's analysis resulted in an appropriate value of 7.5 % for this investigation [51]. A ten-year analysis period is sufficient to prevent an increase in uncertainty.

To improve the reliability and validity of electricity consumption analysis, it is crucial to address the most common outputs of energy evaluators, which include relative errors in reporting, underestimation of energy savings, uncertainty in consumption estimates, variation in accuracy between devices, and dependence on resolution of reporting and sampling rates. These issues can be addressed further in future research. However, since all the data was obtained from reliable sources and real measurements, the study's replicability and reproducibility are guaranteed.

### 2.1 Improvements made to the supermarket lighting

The reform of supermarket lighting included implementing:

• Light-on systems that rely on person detection. These systems utilize technologies such as visible light sensing (VLS) and human motion tracking to control lighting based on human presence and activity. It can automatically adjust illumination and color temperature depending on detected human activities. The components necessary components for detecting and tracking human locations include sensors like photodiodes and LEDs, depth cameras, and thermal cameras. These systems analyze human movements and locations to provide appropriate lighting conditions, energy savings, and enhanced safety in various environments, including smart homes, industrial settings, and public spaces. The use of these systems unnecessary energy consumption by turning off lighting when there are no people in the area.
• Photoelectric cells, also known as photocells, are electronic devices sensitive to light that convert changes in illumination intensity into electrical currents. They consist of a photosensitive metal plate (emitter) and a collector connected to an external circuit. When light of a suitable wavelength hits the emitter, photoelectrons are emitted and drawn to the collector, generating a photo current. As with the previous systems, the goal is to avoid using artificial lighting at times when it is unnecessary. By utilizing photoelectric cells in areas closest to windows or areas with natural lighting, unnecessary use of lighting can be avoided.
• Lighting controllers for advertising signs are designed to adjust the on period and regulate the brightness of outdoor lights based on ambient light intensity or time control. Light detectors can also be used to avoid unnecessary lighting of signs, particularly in spring and summer.
• LED lighting is a rapidly developing and highly energy-efficient technology that has revolutionized the lighting industry. LEDs are semiconductor light sources that emit light when activated. The installation replaced all fluorescent and incandescent lighting with LED technology while maintaining the criteria of light intensity and using LED luminaries of proven quality.

### 2.2 Improvements made to the air conditioning and ventilation facilities of the supermarket

The supermarket's air conditioning and ventilation facilities underwent the following reforms:

• Air conditioning temperature control. Controlling the temperature of the air conditioner involves setting the desired temperature in the supermarket. Proper regulation of temperature, humidity, and outside air intake can lead to significant energy savings. It is important to note that even a one-degree Celsius variation in the programmed comfort temperature in HVAC equipment can result in an increase in electricity consumption between 5 and 8% [52].
• Use of inverter technology. The air conditioners that use inverter technology employ a variable-frequency drive to control the speed of the compressor motor. This technology is known for its ability to self-regulate and maintain a stable temperature by modulating the compressor speed. HVAC equipped with inverter technology avoids the need to start and stop traditional equipment, thereby reducing the energy consumption of the compressors. This equipment enables continuous regulation of its operation based on the temperature programmed for the supermarket. Its use also provides a greater sense of comfort as the temperature is maintained more stably. According to Ref. [53], savings of over 30 % can be achieved with this equipment. Furthermore, it maintains its performance more consistently even at lower air intake temperatures. The drawback of Inverter technology is that it increases the cost of the equipment.
• Free-cooling. This is a design for a cooling system that utilizes colder ambient air to reduce or eliminate the need for chiller or compressor operation. It involves using low external temperatures as a natural cooling source, allowing water chillers or industrial air conditioning systems to bypass mechanical cooling partially or fully. By taking advantage of this technology, part of the energy consumption used in refrigerating the supermarket was avoided by using outside air. The temperature sensors, located both outside and inside the premises, electronically controlled the air intake. Additionally, the inlet air was filtered to remove any potential contaminants.

### 2.3 Improvements made to the refrigeration facilities of the supermarket

**Fig. 1:**
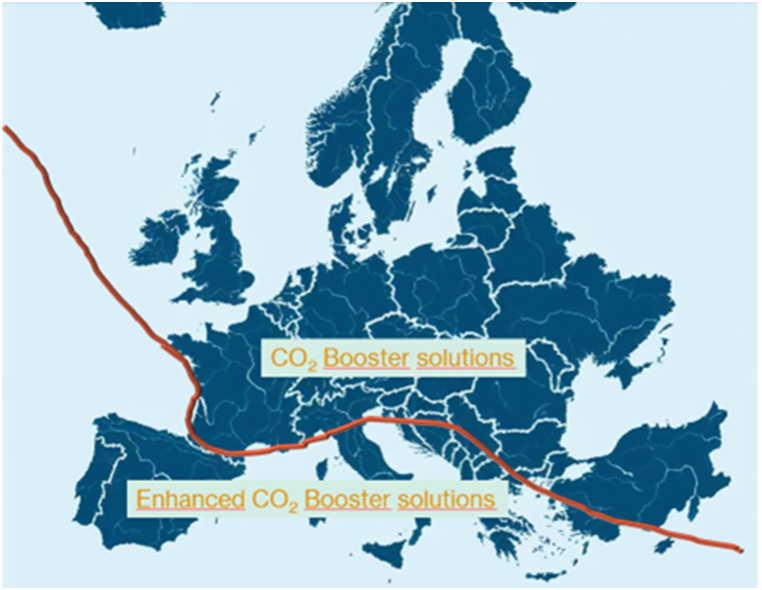
Types of CO_2_ refrigeration systems in Europe.

In Europe, supermarkets traditionally used R12, a CFC, and R502, a CFC/HCFC blend, as refrigerants. However, it is important to note that these refrigerants have negative environmental impacts. To address the issue of ozone depletion, most manufacturers have switched to either R404A, an HFC blend, or R134a. The refrigerants used by the refrigeration equipment before the reform were R134a (for cooling system temperatures above 0 °C) and R404A (for cooling system temperatures below 0 °C). After the reform, R744 was adopted. Refrigeration technology using CO_2_ was analyzed in all European supermarkets. CO_2_ booster solutions were implemented in the northern and central regions of Europe, while enhanced CO_2_ booster solutions were used in the Mediterranean and southern areas of Europe, as shown in Fig. 1. The booster system comprises two stages of refrigerant compression and combines subcritical and transcritical cycles. The technical enhancements made to the CO_2_ system have increased its efficiency when operating at temperatures that may exceed the critical temperature of CO_2_ (31.1 °C), particularly in southern European countries.

Low Global Warming Potential (GWP) refrigerants are being increasingly used in European supermarkets to reduce environmental impact. In addition to the widely used R744, other low-GWP refrigerants are also utilized [[54], [55], [56], [57]]:

• Hydrocarbons, such as propane (R290) and isobutene (R600a). They are commonly used in secondary circuits and cascade systems in supermarkets. Their use is limited to hermetic refrigeration circuits with small loads when the equipment is in enclosed spaces due to their flammability. It is important to comply with established safety standards.
• HFOs (Hydrofluoroolefins). Supermarkets commonly use HFOs such as R448A, R449A, and R513A as refrigerants due to their zero ODP (ozone depletion potential) and low GWP.
• R-717 (ammonia). It is also a suitable refrigerant for both new and existing equipment, with a GWP and ODP of 0. However, its toxicity and slight flammability make it unsuitable for retrofitting systems with existing fluorinated refrigerants. In supermarket refrigeration, R-32 is commonly used in indirect systems due to its classification as ‘low flammability’ and ‘high toxicity’ [58].

The reform of supermarket refrigeration included the following measures:

• Supermarket refrigeration systems utilize enhanced CO_2_ booster solutions with parallel compression and supercharged evaporators featuring two-phase ejectors. This system provides excellent thermodynamic performance at high heatsink temperatures. The use of supercharged evaporators resulted in potential reductions in total irreversibilities that were 5 % lower compared to parallel compression and 22 % lower than basic CO_2_ refrigeration units [59]. In Sweden, a study compared CO_2_ transcritical systems to HFC systems and found that CO_2_ systems have a higher coefficient of performance (COP) and can use 20 % less energy than typical HFC systems [60]. The enhanced CO_2_ booster system sets the pressure in the refrigerant tank is set independently of the outlet conditions of the R744 gas-cooler, allowing for the generation of a biphasic state (liquid-vapor) by setting the pressure below the critical point. Compared to the traditional CO_2_ booster system, the decrease in tank pressure results in a larger increase in enthalpy in the evaporators. This reduces the required flow of refrigerant to achieve the same cooling power and decreases the workload on the compressors. Additionally, cooling is achieved in the suction line of the high compressor, which is significant because practically saturated steam is injected. In this case, it is necessary to evacuate the steam from the tank to prevent an increase in pressure. Automatic regulation is crucial for these devices. A controller must be installed to manage the gradual evacuation of steam, maintaining the pressure around a set value. The transition from transcritical conditions to critical conditions must also be subject to automatic control. The evacuated steam is directed through a pressure reducing valve and injected into the suction line of the high compressor at a pressure equivalent to that of medium temperature service. It is important to consider the potential overheating of the steam in the suction line in this type of installation, as it can contribute to the overheating of the compressed mixture in the high-pressure compressor and increase the total operating cost. In contrast, Scandinavian countries typically use the CO_2_ booster system for direct expansion at low and medium temperatures. This system incorporates a parallel compressor, CO_2_ subcooler, DHW generation, and necessary controls, sensors, and actuators.
• The use of refrigerated cabinets that are closed with glass doors results in significant energy savings. According to sources [38,61], in Mediterranean climate areas and high temperatures, the energy consumption of the refrigeration system compressors can decrease by 25–50 % in the unloaded state. Additionally, this measure can help keep the temperature of the supermarket more stable in winter by avoiding cold areas with open furniture, which can increase the energy consumption of the air conditioning system.

### 2.4 Improvements achieved using residual energy

#### 2.4.1 Industrial cooling installation

**Fig. 2:**
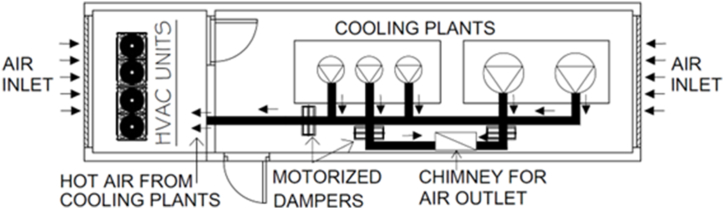
Location of the elements for taking advantage of the residual heat from refrigeration in the air conditioning and industrial cooling machine rooms.

The residual heat evacuated by the condensers of the industrial cooling installation cooling installation was utilized. The main uses of the heat generated during the thermodynamic processes in refrigeration installations are as follows:

• Defrosting in cabinets and freezers using hot gas from compressor discharge eliminates the need for electrical resistors to remove ice from evaporators due to ambient humidity condensation. This reduces energy expenditure by avoiding periodic energy consumption by resistors to transform it into heat for the essential defrost process, preventing evaporator malfunction.
• In industrial cooling processes, excess hot air is recovered to improve the performance of air conditioning systems and reduce energy consumption. Fig. 2 shows the design for the placement of motorized dampers in the hot air discharge ducts of the refrigeration system's condensing units. These dampers are equipped with an opening control that depends on the temperature of the air conditioning machine room. Thus, the refrigeration system introduced hot air when the temperature of the air conditioning machine room dropped below 10 °C. The dampers automatically close to interrupt this air intake when the room reaches 10 °C. This improvement prevents condensation in the refrigeration system and improves the air intake conditions of the HVAC units in winter. This increased the coefficient of performance (COP) of the HVAC system, resulting in reduced electrical consumption of the heat pumps.

#### 2.4.2 Bakery facilities

**Fig. 3:**
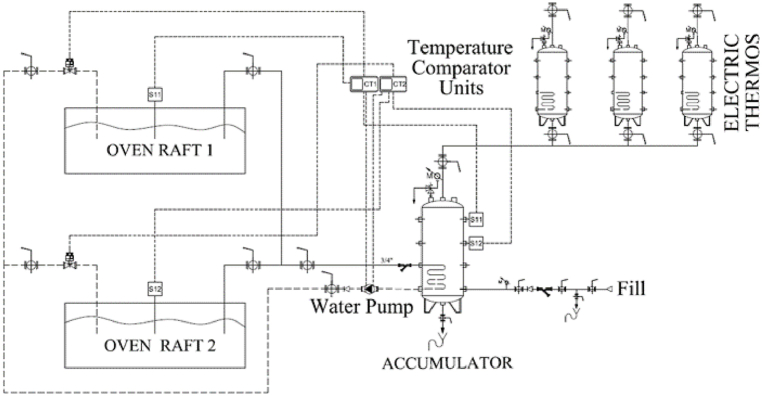
IWERS circuit diagram.

The reform in the bakery facilities of the supermarket included an incorporation of the integrated waste energy recovery system (IWERS) in the electric bread ovens. Through this system, which is described in Ref. [62] and that uses a water tank equipped with a coil, electric thermos, a water pump and temperature comparator units among other elements, the residual heat from the condensation of bread ovens is used. The thermal energy generated during the manufacturing processes of bakery products in the ovens is recovered and used to heat domestic hot water (DHW) in supermarkets. Fig. 3 shows a scheme of the main system. In our analysis, this system was used in two ovens in a supermarket similar to the one analyzed. The IWERS reduces both the consumption of electrical energy used to obtain DHW and the consumption of clean water used in the condensation of the steam generated in the ovens.

After applying improvements in energy efficiency and the use of residual energy, consumption data from various areas of commerce became obtainable. As previously stated, the analyzed supermarket shares the same configuration and average size as the most significant supermarket companies operating throughout Europe. Although climatic conditions may influence supermarket air conditioning usage, the majority of energy consumption (88–91 %) comes from refrigeration of products in refrigerated or freezer cabinets, lighting that remains on for most of the day, and ovens and bakery proofers. It is important to note that this energy consumption is common throughout Europe [17,35,37,40,62].

## 3 Results

Table 5 in the annex provides information on electricity prices for non-household consumers in European countries, according to Ref. [7]. The table displays the average price for the 27 EU countries and the 19 countries in the euro area. It includes half-yearly average prices (S) from 2018 to the end of 2021, applicable to electricity consumed in European supermarkets. The years 2018 and 2019 are considered the pre-Covid period. In 2020, Europe experienced widespread lockdowns due to the Covid-19 pandemic, making it a crucial period for the disease. In 2021, the de-escalation of Covid-19 restrictions began, and the European population was no longer subject to general confinement. This marked the beginning of the post-Covid period, particularly in the second semester.

### 3.1 Distribution of electricity consumption in a post-Covid period analyzed and unrefurbished supermarket

Table 1 displays the results obtained from the analysis of an unrefurbished supermarket during the pre-Covid stage. An unrefurbished supermarket is an old supermarket that has not undergone any improvements or adaptations to its construction or facilities since its construction. The results of both investigations mentioned in section 1.2 were consistent with the percentages reflected in Table 2. The slight variations observed with increases in refrigeration or industrial cooling may be attributed to the growing trend of commercializing refrigerated products, as well as the slight increase in the production of bakery items.

**Table 1:** Distribution of annual electrical consumption per m^2^ of useable surface area of unrefurbished supermarket and percentages.

	Annual consumption/useful area (kWh/m^2^·year)	Percentage (%)
Lighting	129.47	27.98
Refrigeration	229.83	49.68
HVAC	45.12	9.75
Bakery	44.11	9.53
Hot Water	1.68	0.36
Plugs	7.89	1.72
Others	4.55	0.98

Additionally, this report will reflect the results of the energy improvements made to various supermarket facilities. Electricity consumption data was collected over four years in six-month intervals to account for seasonal and social variations in the consumption needs of lighting and thermal installations, as well as in the production and sale of products.

### 3.2 Reform of the lighting installation

**Fig. 4:**
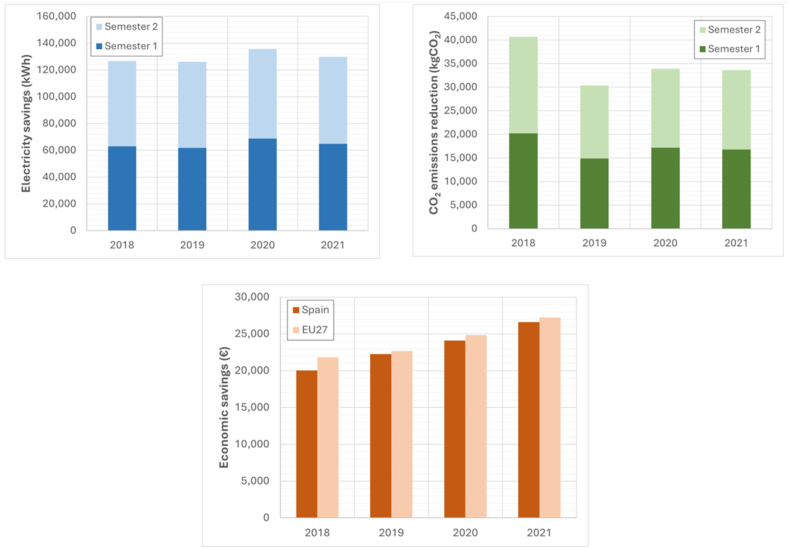
Savings achieved through the reform of the lighting installation.

Fig. 4 shows the economic concepts of annual energy consumption savings, economic savings, and savings in associated polluting emissions resulting from the lighting installation improvements. The results were obtained for the two semesters of 2018, 2019, 2020 and 2021, based on the energy prices provided by Eurostat [7]. The carbon footprint for energy consumption or carbon dioxide equivalent in Spain was 0.321 kg CO_2eq_/kWh in 2018, 0.241 kg CO_2eq_/kWh in 2019, 0.250 kg CO_2eq_/kWh in 2020 and 0.259 kg CO_2eq_/kWh in 2021. These figures depend on the mix of the Spanish electricity network published by the Spanish National Markets and Competition Commission (CNMC) [63]. In comparison, the average footprint in the EU27 during 2021 was 0.2307 kg CO_2eq_/kWh according to data from the European Environment Agency, which is dependent on the EU [64].

### 3.3 Reform of air conditioning and ventilation facilities

**Fig. 5:**
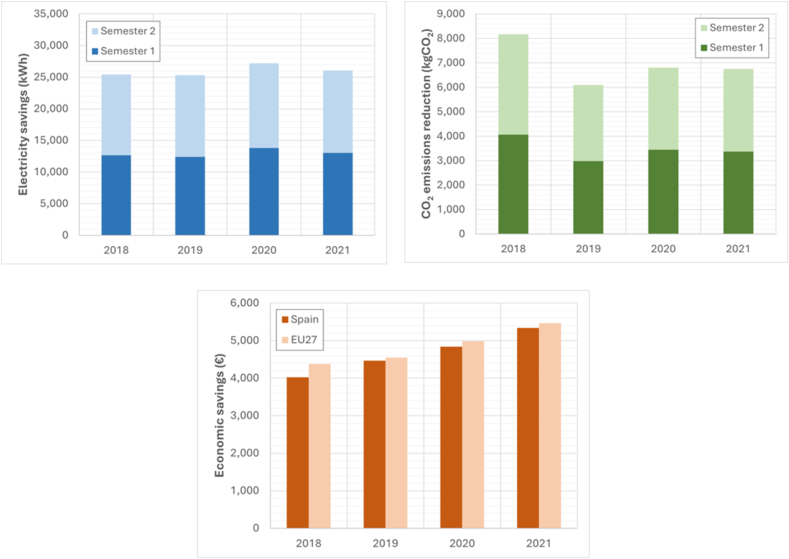
Savings achieved through the reform of the HVAC installation.

Fig. 5 displays the economic concepts of annual energy consumption savings, economic savings, and savings in associated polluting emissions resulting from improvements in air conditioning and ventilation installation.

### 3.4 Reform of the refrigeration system

**Fig. 6:**
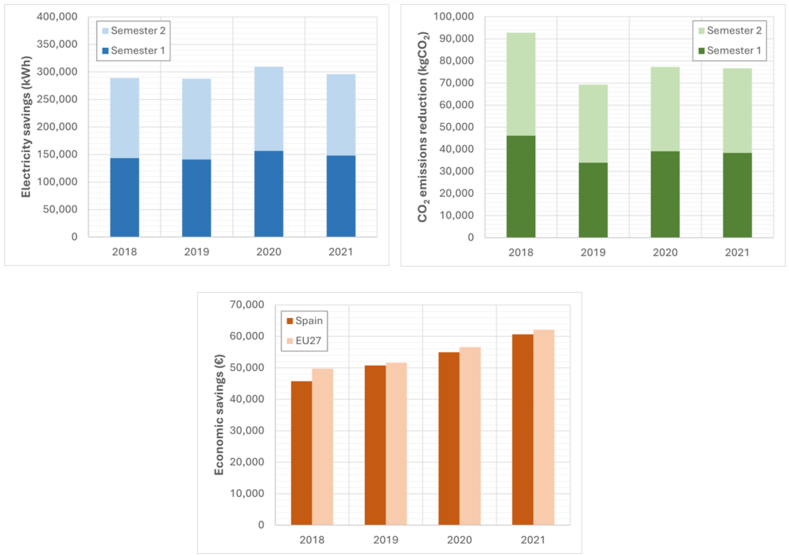
Savings achieved by reforming refrigeration installation.

Fig. 6 shows the economic concepts related to annual energy consumption savings, economic savings, and savings in associated polluting emissions that can be achieved through improvements in refrigeration facilities.

### 3.5 The utilization of thermal energy or residual heat

Residual thermal energy generated by supermarket activity was utilized in two areas of commerce.

#### 3.5.1 Residual heat generated by bakery ovens

The IWERS not only reduced the consumption of electrical energy used to obtain DHW, but also achieved a significant reduction in the consumption of clean water used in the condensation of steam generated in the ovens.

#### 3.5.2 Energy use of residual hot air from cooling plants

**Fig. 7:**
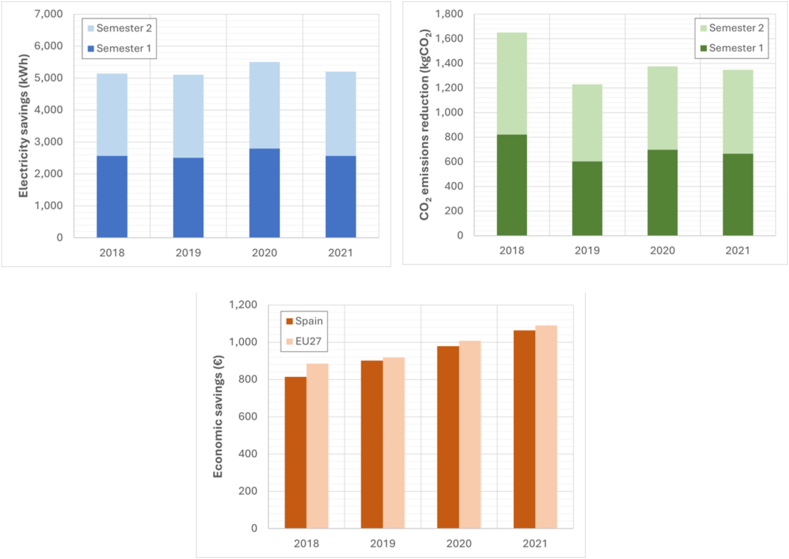
Savings achieved using thermal energy or residual heat.

This system was particularly recommended during the coldest times of the year, resulting in energy consumption savings of over 20 %. When the outside temperature was higher and it was unnecessary to supply hot air to the room where the cooling plants were located, the motorized gates remained closed, preventing the circulation of hot air from the cooling plants to the air conditioning room. The system of thermal energy use made it possible to reduce the HVAC's annual electrical energy consumption by over 10 %. Fig. 7 shows the economic concepts of annual energy consumption savings, economic savings, and savings in associated polluting emissions, which are achieved through the residual heat generated in the supermarket.

### 3.6 Embodied carbon associated with the application of energy improvements

In the context of supermarket reform, ‘embodied carbon’ refers to the carbon emissions resulting from the energy and materials used in constructing, renovating, or operating supermarkets. The embodied carbon associated with the improvements made to the supermarket can be analyzed through three technological advancements:

• Scholland & Dillon's research [65] shows that the GWP for LED lighting, including raw materials, manufacturing, transportation, and disposal, is 54.98 kg CO_2eq_ per square meter of supermarket.
• Additionally, utilizing residual heat from two bakery ovens during installation resulted in a GWP of 1800 kg CO_2eq_, as calculated using software [66].
• The installation of commercial refrigeration would decrease its carbon footprint by using R744 as a refrigerant, which has a GWP of 1, compared to traditional refrigerants such as R134a or R404A, which have GWPs of 1430 and 3920, respectively [58].

### 3.7 Annual savings resulting from energy improvements and the corresponding amortization periods for the necessary investments

Table 2 presents a comparison analysis of the energy savings achieved through various measures aimed at improving the energy efficiency of the supermarket. The energy savings achieved are expressed per square meter of supermarket surface.

**Table 2:** Energy savings achieved by implementing measures to improve the supermarket's energy efficiency.

Applied measure	Energy saving (kWh/year·m^2^)
Reform of the lighting installation	91.35
Reform of air conditioning and ventilation facilities	22.33
Reform of the refrigeration system	99.83
Use of thermal energy or residual heat	4.53

The potential savings observed in Spanish supermarkets can be applied to the entire EU27 due to their similar socioeconomic characteristics. Therefore, assuming a population of 47,435,597 in Spain and 447,135,481 in the EU27 in 2022 [67,68], the proposed energy-saving measures are estimated to result in the savings detailed in Table 3 for all existing supermarkets.

**Table 3:** Savings achieved by reforming Spanish and European supermarkets.

	Energy savings (TWh/year)	Economic savings (M€/year)	Annual savings in CO_2_ production (t CO_2_/year)
**Spain**	11.214	2297.23	290.45 × 10^7^
**UE27**	105.707	22,161.41	243.87 × 10^8^

**Fig. 8:**
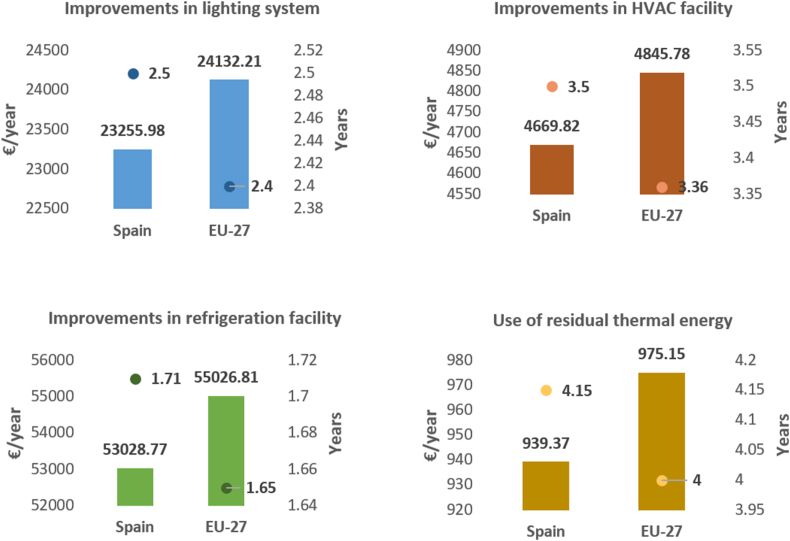
Energy saving measures in supermarkets: Average annual financial savings and investment payback periods from 2018 to 2021 (€/year).

The supermarket invested in several improvements (C), including lighting (57,920.50 €), HVAC facilities (11,700 €), refrigeration facilities (90,850 €), and the use of thermal energy or residual heat (3900 €). Fig. 8 displays the average annual financial savings (S) from 2018 to 2021 and the investment amortization periods (A) for each improvement.

## 4 Conclusion

The primary approach to mitigate the impact of rising electricity costs in supermarkets should focus on enhancing facility energy efficiency by utilizing residual heat.

The measures that yielded the greatest savings were those implemented in the refrigeration system, which also had the shortest investment payback period (less than 2 years).

The measures that yielded the second best results were those applied to the lighting installation, with savings half of those achieved in refrigeration but still significant. These measures also had short payback periods, not exceeding 2.5 years.

The third most important measures were those applied to HVAC systems, with payback periods one year longer than those for lighting, not exceeding 3.5 years.

The measures applied to bakery ovens presented the longest payback periods, at 4.5 years, and the lowest savings. However, with a reduced investment of less than 1 €/m^2^ of the useful surface area of the establishment, they are the most economical measures to apply.

All proposed measures had short periods of amortization for the economic increase that the implementation of the improvements entails, with none exceeding 5 years. Therefore, they are highly recommended from an economic standpoint.

The cost of energy is projected to increase, resulting in even shorter amortization periods.

The most effective measures for maximizing environmental benefits of the investment were improvements in refrigeration, accounting for 65 % of the total reduction in emissions, followed by improvements in lighting installation at 28 %, HVAC at 6 %, and finally, bakery improvements at 1 % of the total.

The research shows that energy savings lead to an improvement in supermarkets' income statements. In addition, environmental improvements are associated with the reduction of polluting emissions related to energy consumption.

Data usability:

• Researchers can use the method employed in their research, research findings, or conduct new research based on this study. For example, they could analyze the results obtained with alternative low GWP refrigerants besides CO_2_. The ‘discounted payback period approach’ is suggested as a potential area for future research. This method calculates the number of years required to recoup the initial investment for a project, considering the time value of money. It is important to note that the ‘simple payback period’ does not consider the time value of money. The formula and approach for the discounted payback period are similar to those of the simple payback period, but present value factors must be applied to any future cash flows.
• The results obtained can inform policies aimed at enhancing efficiency in commercial facilities, thereby reducing electricity consumption and polluting emissions in society. Additionally, it is important to adhere to conventional academic structure and formatting, including consistent citation and footnote style. It is important to note that any proposed measures should be evaluated objectively and implemented only if they are proven to be effective. The language used should be clear, concise, and free from any biased or emotional language. Finally, the text should be free from any grammatical errors, spelling mistakes, and punctuation errors. Economic improvements in the EU27 were 3.8 % higher than in Spain during the four-year period analyzed.
• Industrial companies can use both energy and economic data to plan investments and implement policies that improve energy efficiency in their businesses.

Perspectives:

• In Europe and worldwide, policies are being implemented to reduce energy consumption in commerce. Economic incentives for entrepreneurs in the commercial sector are among these measures. Therefore, the potential for implementing energy-saving measures and improving efficiency in European supermarkets is promising.
• Additionally, European supermarkets are increasingly focused on reducing energy consumption and increasing efficiency. Various European legislations aim to reduce polluting emissions and the high cost of energy. These efforts present an opportunity to generate substantial cost savings and environmental benefits.

Perspectives and future research:

• A limitation of the study is that it did not assess the impact of the proposed measures for different sizes of supermarkets. This could be a new line of research.
• Additionally, more in-depth statistical analyses of the results obtained with the improvements adopted should be carried out in future research.
• In future research, it may be necessary to consider adapting the measures to future technological improvements that reduce energy consumption in existing supermarket facilities.

## Ethics declarations

Informed consent was not required for this study because the data used in this study came from public databases, and did not involve animal and human experiments, as well as other data related to human privacy.

## Data availability statement

Data included in article/supp. material/referenced in article. The raw/processed data required to reproduce the above findings cannot be shared at this time due to legal/ethical reasons.

## CRediT authorship contribution statement

**Juan Carlos Ríos-Fernández:** Writing – review & editing, Writing – original draft, Visualization, Validation, Supervision, Software, Resources, Project administration, Methodology, Investigation, Funding acquisition, Formal analysis, Data curation, Conceptualization. **Juan Manuel González-Caballín:** Writing – original draft, Visualization, Data curation. **Andrés Meana-Fernández:** Writing – review & editing, Validation, Data curation. **Antonio José Gutiérrez-Trashorras:** Writing – review & editing, Writing – original draft, Validation, Supervision.

## Declaration of competing interest

The authors declare that they have no known competing financial interests or personal relationships that could have appeared to influence the work reported in this paper.

## Figures and Tables

**Table 4:** Formats of supermarkets according to their surface [[31], [32], [33]].

Type of supermarket	Exhibition and sales area (m^2^)	Commercial area in 2020 (%)
Small supermarket	100–399	12
Medium supermarket	400–999	36
Large supermarket	1000–2499	32
Hypermarket	>2500	20

**Table 5:** Electricity prices for non-household consumers in European countries. Average price for 27 countries in the EU and 19 countries in the euro area (€/kWh).

	S1 2018	S2 2018	S1 2019	S2 2019	S1 2020	S2 2020	S1 2021	S2 2021
**Denmark**	0.2807	0.2787	0.2628	0.2604	0.2524	0.2622	0.2900	0.3291
**Germany**	0.2231	0.2256	0.2298	0.2232	0.2404	0.2378	0.3193	0.2556
**Italy**	0.1968	0.1999	0.2237	0.2213	0.2057	0.1992	0.2259	0.2485
**Portugal**	0.1835	0.1839	0.1703	0.1807	0.1745	0.1716	0.2089	0.1844
**Cyprus**	0.1794	0.2201	0.2075	0.2192	0.1928	0.1661	0.1976	0.2303
**Ireland**	0.1772	0.1823	0.1843	0.1826	0.1886	0.1860	0.2555	0.2375
**Netherlands**	0.1737	0.1277	0.1654	0.1291	0.1796	0.1399	0.1281	0.1620
**Belgium**	0.1727	0.1841	0.1923	0.1848	0.1872	0.1821	0.2702	0.2159
**Greece**	0.1632	0.1627	0.1569	0.1534	0.1492	0.1536	0.1680	0.2556
**Slovakia**	0.1617	0.1650	0.1768	0.1812	0.1849	0.1856	0.1668	0.1900
**Malta**	0.1596	0.1600	0.1593	0.1596	0.1593	0.1595	0.1279	0.1594
**Latvia**	0.1570	0.1560	0.1567	0.1583	0.1539	0.1563	0.1403	0.1880
**France**	0.1514	0.1440	0.1568	0.1519	0.1702	0.1515	0.1403	0.1542
**Czechia**	0.1507	0.1565	0.1661	0.1716	0.1735	0.1680	0.1802	0.1674
**Austria**	0.1463	0.1480	0.1522	0.1514	0.1614	0.1624	0.2216	0.1796
**Spain**	0.1445	0.1718	0.1791	0.1741	0.1767	0.1790	0.2323	0.1774
**Poland**	0.1404	0.1403	0.1502	0.1335	0.1616	0.1599	0.1548	0.1697
**Croatia**	0.1293	0.1294	0.1387	0.1430	0.1385	0.1352	0.1291	0.1530
**Slovenia**	0.1289	0.1292	0.1380	0.1812	0.1402	0.1419	0.1662	0.1440
**Hungary**	0.1253	0.1240	0.1415	0.1400	0.1443	0.1396	0.1003	0.1489
**Montenegro**	0.1150	0.1131	0.1223	0.1235	0.1259	0.0978	0.0980	0.0974
**Estonia**	0.1148	0.1243	0.1198	0.1180	0.1063	0.1135	0.1324	0.1974
**Lithuania**	0.1134	0.1246	0.1252	0.1275	0.1298	0.1386	0.1348	0.1908
**Bulgaria**	0.1130	0.1162	0.1194	0.1136	0.1117	0.1150	0.1024	0.1853
**Romania**	0.1119	0.1161	0.1275	0.1136	0.1422	0.1389	0.1536	0.1643
**Luxembourg**	0.1118	0.1147	0.1199	0.1201	0.1269	0.1267	0.1988	0.1323
**Moldova**	0.1089	0.1038	0.1005	0.1036	0.1269	0.1267	0.0851	0.0810
**Finland**	0.1033	0.1077	0.1092	0.1093	0.1068	0.1130	0.1767	0.1160
**Bosnia and Herzegovina**	0.1016	0.0981	0.1029	0.1027	0.1061	0.1061	0.0875	0.1041
**Kosovo**	0.1013	0.1018	0.0874	0.0881	0.0878	0.1061	0.0605	0.0889
**Serbia**	0.1002	0.1032	0.1124	0.1142	0.1135	0.1109	0.0791	0.1227
**North Macedonia**	0.0997	0.1025	0.1051	0.1073	0.1087	0.1071	0.0841	0.1935
**Sweden**	0.0993	0.1068	0.1131	0.1035	0.1090	0.1032	0.1791	0.1497
**Norway**	0.0986	0.1117	0.1051	0.0969	0.0640	0.0610	0.1826	0.1489
**Iceland**	0.0919	0.0878	0.0888	0.0893	0.0640	0.0816	0.1356	0
**Ukraine**	0.0752	0.0779	0.0822	0.0893	0.0759	0.0686	0.0485	0
**Turkey**	0.0727	0.0754	0.0891	0.1023	0.0997	0.0829	0.0834	0.087
**Georgia**	0.0645	0.0743	0.0781	0.0625	0.0660	0.0579	0.0631	0.0808
**Albania**	0	0	0.1248	0	0	0	0	0
**Liechtenstein**	0	0	0	0	0	0	0.2071	0
**Euro area (19 countries)**	**0.1809**	**0.1818**	**0.1927**	**0.1865**	**0.1961**	**0.1882**	**0.2322**	**0.2074**
**European Union (27 countries)**	**0.1715**	**0.1727**	**0.1830**	**0.1766**	**0.1865**	**0.1797**	**0.2192**	**0.2001**
